# Anemonefish, a model for Eco-Evo-Devo

**DOI:** 10.1186/s13227-020-00166-7

**Published:** 2020-10-07

**Authors:** Natacha Roux, Pauline Salis, Shu-Hua Lee, Laurence Besseau, Vincent Laudet

**Affiliations:** 1 Sorbonne Université, CNRS, UMR « Biologie Intégrative Des Organismes Marins », BIOM, 1, 66650 Banyuls-sur-Mer, France; 2 Lab of Marine Eco-Evo-Devo, Marine Research Station, Institute of Cellular and Organismic Biology, Academia Sinica, Taipei, Taiwan; 3 Marine Eco-Evo-Devo Unit, Okinawa Institute of Science and Technology, 1919-1 Tancha, Onna son, Okinawa, 904-0495 Japan

**Keywords:** Anemonefish, *Amphiprion*, Eco-evo-devo

## Abstract

Anemonefish, are a group of about 30 species of damselfish (Pomacentridae) that have long aroused the interest of coral reef fish ecologists. Combining a series of original biological traits and practical features in their breeding that are described in this paper, anemonefish are now emerging as an experimental system of interest for developmental biology, ecology and evolutionary sciences. They are small sized and relatively easy to breed in specific husbandries, unlike the large-sized marine fish used for aquaculture. Because they live in highly structured social groups in sea anemones, anemonefish allow addressing a series of relevant scientific questions such as the social control of growth and sex change, the mechanisms controlling symbiosis, the establishment and variation of complex color patterns, and the regulation of aging. Combined with the use of behavioral experiments, that can be performed in the lab or directly in the wild, as well as functional genetics and genomics, anemonefish provide an attractive experimental system for Eco-Evo-Devo.

## Natural habitat and life cycle

Anemonefish are protandrous hermaphrodites that belong to the Pomacentridae family, known as damselfish. Within the 300 species of this family, the 30 species of anemonefish are clustered into two genera: *Amphiprion* and *Premnas*, the latter including a single species [1, 2]. The phylogeny of anemonefish is well resolved with mtDNA and nuclear markers, and more recently with complete genome sequences [3, 4].

**Fig. 1:**
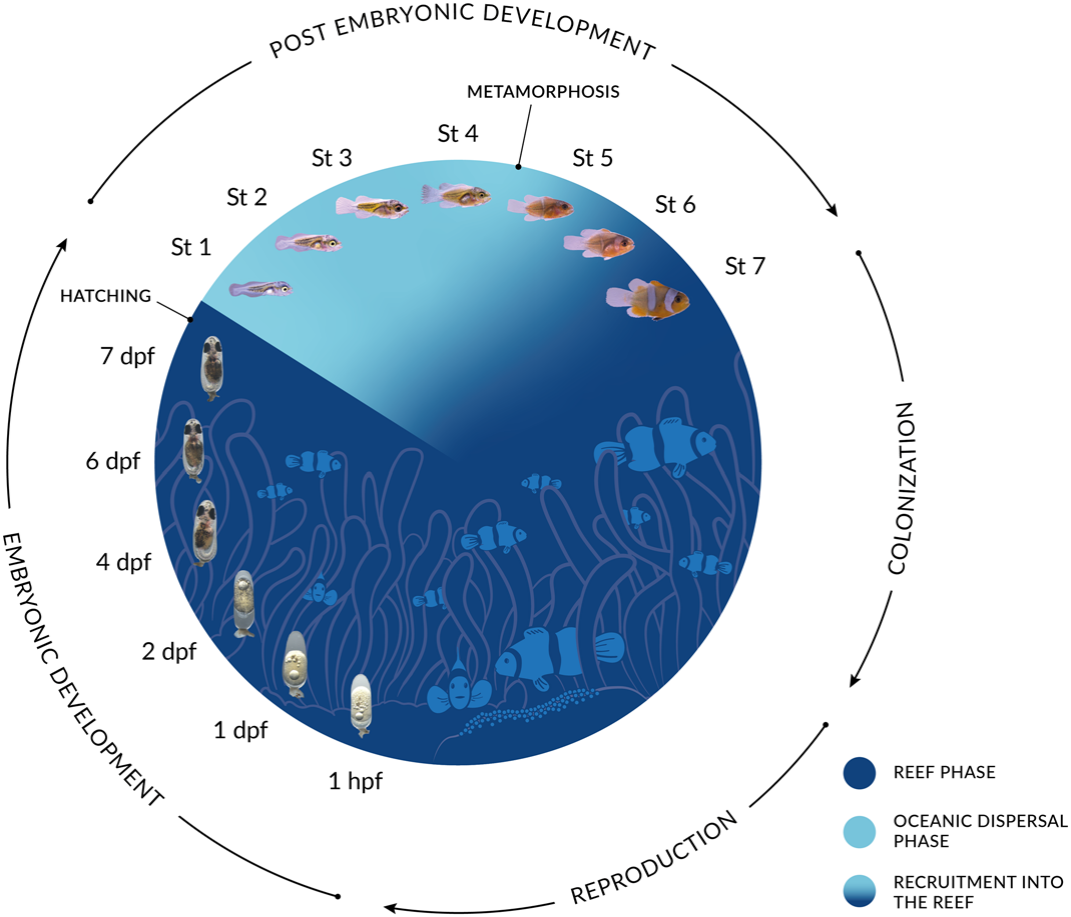
Life cycle of anemonefish. Anemonefish lay their eggs close to their sea anemone, where they will develop for six to ten days, depending on temperature and species. After hatching, larvae are directly dispersed into the open ocean, where they will grow for 10 to 15 days before returning to the reef. Larval development is characterized by seven distinct stages [75]. The transition between the ocean and the reef is associated with the metamorphosis of larvae into juveniles. Juveniles will then settle in a sea anemone. Picture credits: Embryos: Shu-Hua Lee; Larvae: Natacha Roux and Pauline Salis

Living in social groups and in symbiosis with 10 distantly related sea anemone species, anemonefish are found from the Indian Ocean to the western Pacific Ocean with a high concentration of species in the Indo-Malay archipelago [5, 6]. Within their host sea anemone, anemonefish form a colony, which consists of a breeding pair and a variable number of juveniles of smaller size [7]. The breeding pair is composed of a dominant female and a sub-dominant male, which both defend the colony. Every two to 3 weeks, the breeding pair lays between 100 and 500 eggs on the substrate near their host anemone, and provide parental care to the eggs [8]. Parental care consists of fanning the eggs using their pectoral and caudal fins, to increase oxygenation, and removing dead or weakened eggs and particles using their mouth [9]. Most of the parental care are provided by the male [10, 11] but in some species female may participate actively [9, 12]. The sexually immature individuals don’t participate in brood care. They are ranked by order of size and gain access to reproduction only when one member of the breeding pair dies. The social dynamics have mostly been studied in *A. ocellaris* and more studies are needed to fully understand the variation that can occur in other species. After an embryonic development of seven to 10 days (depending on the water temperature and the species), hatching occurs and larvae disperse into the ocean for 10–15 days, which corresponds to one of the shortest oceanic larval duration in coral reef fish (Fig. 1). At the end of this pelagic phase, larvae transform via metamorphosis into small juveniles that will locate a reef and actively look for a host sea anemone using their sensory abilities combining visual, chemical and acoustic cues [13]. During the metamorphosis, larvae lose their larval characteristics (light pigmentation, elongated body shape) and start to resemble miniature adult individuals, with an ovoid body shape and an adult pigmentation pattern which consists in most species of white bars on a bright yellow, red or brown/black background (Fig. 1). Of note, little is known concerning how new recruits are socially accepted in a new colony during recruitment.

## Field collection and lab culture

One of the advantages of working on anemonefish is that post-metamorphosed juveniles and adults can easily be spotted in the field thanks to the symbiosis with sea anemones. Compared to most marine fish, they never leave the immediate vicinity of their habitat (the sea anemone) and are thus particularly suited for individual long-term monitoring. Scuba diving and hand nets are the two major tools required to observe and collect anemonefish in the wild. It is thus easy to take pictures, collect mucus, clip fins (for genetic analysis), tag individuals, conduct behavioral study, and collect embryos, etc. All of these sampling techniques can be done without removing the animal from its natural environment, which is important to consider as these species undergo significant fishing pressure due to the aquarium trade [14]. In contrast to adults, collecting wild anemonefish larvae represents a major difficulty. To our knowledge, usual techniques used to collect fish larvae such as crest nets, light traps and even plankton nets very rarely succeeded to catch larvae, which are mostly described from laboratory raised fish [15]. It is possible to collect eggs spawned by adults close to their sea anemone host but finding larvae in the wild is like looking for a needle in a haystack and still represents a major challenge.

**Fig. 2:**
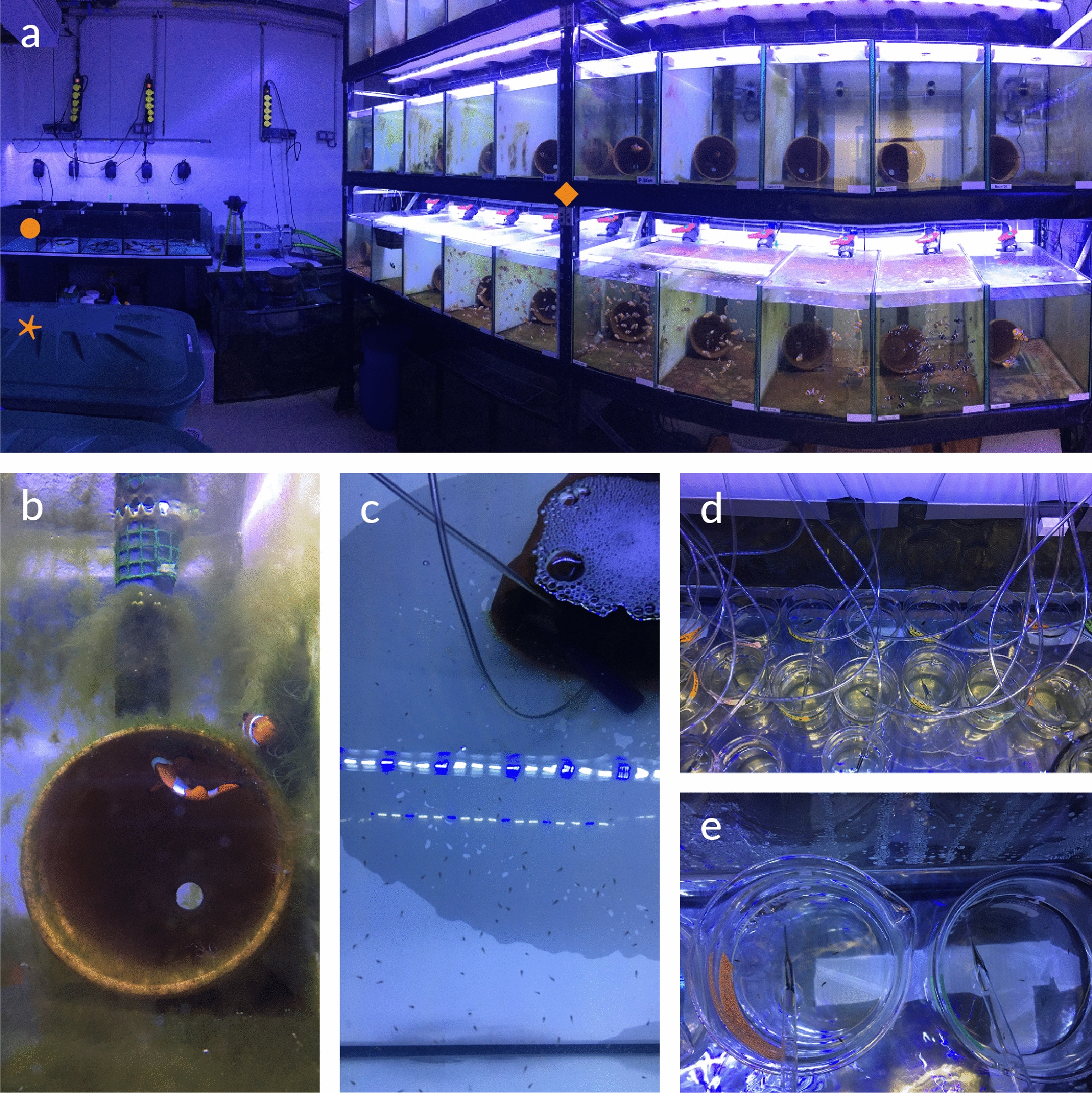
Anemonefish rearing. **a** Picture of the rearing structure of anemonefish in the marine station of Banyuls-sur-mer, with breeding pair tanks and juvenile tanks on the right-hand side (orange square), larval rearing tanks on the left-hand side in the background (orange dot), and artificial seawater reserve tanks on the left (orange star). **b** Close up of an *A. ocellaris* breeding pair and their terracotta pot. **c** close up of larval rearing tank. **d**, **e** Close up of low volume rearing in beakers

As they are one of the first coral reef fish to be reared in aquaria, anemonefish are very well suited for laboratory rearing and experiments. Breeding pairs can be held without sea anemones in 60L tanks filled with artificial or natural filtered seawater (Fig. 2a). To successfully rear anemonefish, it is critical to pay attention to water parameters, such as temperature, salinity and pH (between 8 and 8.5), and to photoperiod [10, 16, 17]. Other parameters such as nutrients (NH3, NO2, NO3-) and food fatty acid contents are also required for breeding pairs health, quality of clutch and larval survival rates [18, 19]. A terracotta pot can be placed in the aquarium as a support for anemonefish clutches (Fig. 2b). The night of hatching, the pot with the clutch is transferred into an independent larval rearing tank before light extinction (Fig. 2c). Larvae are fed with living preys (rotifers, nauplii of *Artemia sp,* and the green algae *Nannochloropsis occulata*) until the completion of metamorphosis and then artificial food is given. It is important to perform daily water changes to remove dead individuals, food waste and feces to ensure the stability of the seawater (Roux et al., unpublished data).

For experimental purposes such as developmental monitoring or pharmacological treatments, small groups of anemonefish larvae can be reared in low volume (500 ml). In such cases, rearing volume might be an issue for treatments lasting several days when the compound is either rare and/or expensive. The low volume rearing protocol consists of an independent system in which 5–10 larvae are introduced in 500 ml glass beakers placed in a warm water bath (same temperature as breeding pair tank) (Fig. 2d). The rearing condition (seawater, photoperiod, food, water changes) remains the same as in a 60L tank (Roux et al., unpublished data).

## Major interests and research questions

### Symbiotic relationship

All anemonefish species have evolved the ability to live in close association with sea anemones that belong to 3 distantly related families (*Thalassianthidae*, *Actinidae*, *Stichodactilidae*) [6]. Studied since the end of the nineteenth century [20], this symbiosis has fascinated scientists for two main reasons. First, anemonefish are protected by their host against predation, by swimming within the tentacles of the anemone, whose discharge of nematocysts can kill other fish. Second, there is a complex species-specificity of this mutualistic relationship between fish and the 10 possible sea anemone hosts, which is probably related to the toxicity levels of the hosts [21]. Recent data have shown that the giant sea anemones that host anemonefish form three independent distantly related groups and that their taxonomy is still unclear [22, 23]. This allows to conclude that, in contrast to anemonefish for which the symbiosis has been established once [4], on the sea anemone side the symbiosis has evolved independently at least three times.

Studies investigating the anemonefish symbiosis have brought new insights into (i) the genetic and biochemical mechanisms developed by anemonefish to avoid the stinging of the sea anemone nematocysts, (ii) the variable host specificity displayed by the different species, and (iii) the changes occurring on the mucus microbiota of the two animals during the initiation of the symbiosis [24–29]. It has been shown that this symbiotic interaction has been critical for the evolutionary radiation of anemonefish [3, 4]. In contrast, the diversification of giant sea anemones predates the symbiosis [22, 23]. The taxonomy of the sea anemones is far from settled and will be important to better understand this complex relationship.

### Life span

The lifespan of fish spans more than two orders of magnitude, from a few weeks in gobies and a few months in annual killifish to 9 years for the damselfish *Chromis chromis*, and up to several centuries in the Greenland shark [30–33]. The lifespan of *A. percula* has been estimated to be about 30 years [34]. Sahm and collaborators (2019) performed a survey of anemonefish lifespan data in the wild and in captivity, in which they identified at least two additional species (*A. ocellaris* and *A. melanopus*) with a lifespan of over 20 years [35]. Because this lifespan is longer than other teleost fish of the same size, anemonefish could become an experimental model for long-lived vertebrates [34]. For example, recently, anemonefish have been used to understand the genetic architecture underlying differences in lifespan [35]. Taking advantages of the transcriptome of 5 anemonefish species, Sahm et al. (2019) identified genes that underwent accelerated molecular evolution in association with the evolution of a long lifespan [35].

### Social control of size hierarchy

Within each colony of anemonefish, there is a dominant female, the largest fish, a sub-dominant male, second largest fish, and smaller sexually immature individuals ranked by size [7]. The size hierarchy represents a queue to attain dominant status and reproduction; individuals only ascend in rank when a higher ranked individual disappears, and the smallest fish in the group is always the youngest recruit. Anemonefish could provide an experimental system for understanding the ultimate and proximate causes of size hierarchy. Moreover, very little is known concerning how new recruits are socially accepted in a new colony during recruitment. The role of hormones in eliciting the dominant and subordinate specific features in behavior or pigmentation and how environmental changes might influence these hierarchies are promising research avenues that can be addressed using this model in which the female is the dominant sex.

### Sex change

The plasticity of sex is frequent in teleosts and is made possible by an extraordinary plasticity of gonadal development and sexual expression [36, 37]. An illustration of this plasticity is successive hermaphroditism, presented in at least 27 families, including that of Pomacentridae. The factors triggering sex changes in teleosts may be size dependent or socially mediated [38]. However, the internal mechanisms underlying sex changes remain poorly understood. Given their social organization, their small size and their easy maintenance in aquaria, anemonefish could be relevant models for studying sex change.

Anemonefish are monogamous protandrous (male to female transition) hermaphrodites whose phenotypic sex status is socially controlled within the colony. The removal of the dominant female triggers the sex inversion of the functional male, which in turn induces the differentiation of the biggest immature juvenile into a mature male to form a new breeding pair [39]. In anemonefish, it is easy to experimentally induce sex change, both in the lab and in the wild by simply removing the dominant female. Following the dominant male during its transition to female allows for a better insight into the molecular and physiological mechanisms governing sex change [39–44].

### Color pattern evolution

**Fig. 3:**
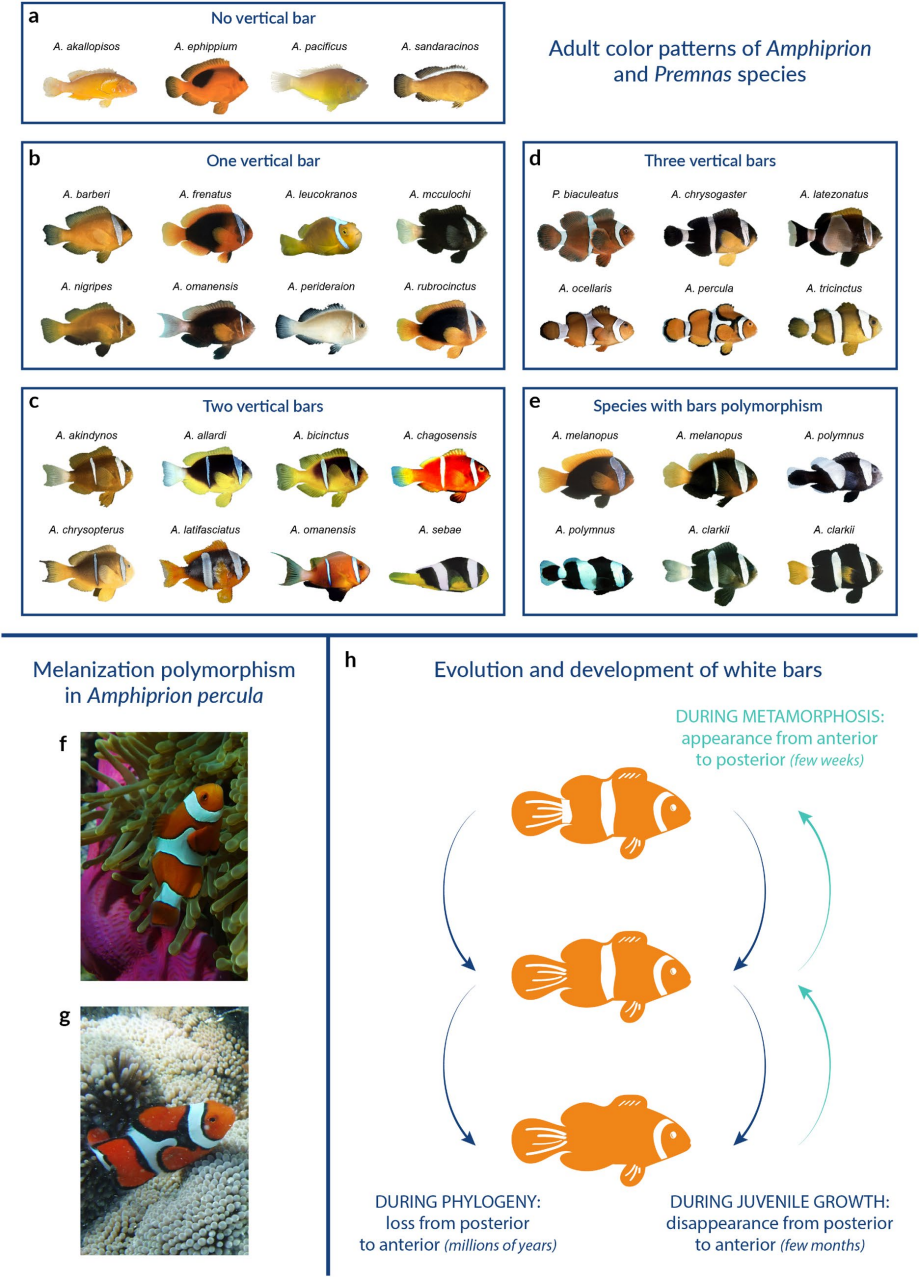
Anemonefish color pattern diversity.** a**–**e** Pictures of adult anemonefish classified according to their color patterns. **a** No vertical bar, **b **One vertical bar on the head, **c** Two vertical bars,**d** Three vertical bars, **e** Fishes having polymorphic bar patterns [47]. **f**, **g** Pictures of wild *Amphiprion percula* adults showing a melanization polymorphism depending on the sea anemones species they have been recruited to, either *Heteractis magnifica* (**f**) or *Stichodactyla gigantea* (**g**)

Whereas most previous investigations of teleost pigment pattern formation focused on the horizontal stripe patterns of the zebrafish (*Danio rerio*), anemonefish offer an alternative case study in which different mechanisms controlling vertical bar patterns are at play and can be deciphered [45, 46]. The 30 anemonefish species display a simple color pattern made of 0 to 3 white bars, containing iridophores, visible on a darker body background (red, orange or black) (Fig. 3a–e). Anemonefish acquire their adult color pattern during post-embryonic development and white bars appear in a stereotypic manner from the anterior to the posterior part of the body [47].

Recently, the occurrence of these bars has been mapped on the anemonefish phylogeny to reconstruct the ancestral states in terms of white bar presence/absence [47]. Through this analysis, it has been shown that the diversification of the anemonefish color pattern results from successive caudal to rostral losses of bars during evolution. The juveniles of some species have supplementary bars that later disappear in a caudo-rostral sequence. The reduction of bar number during the ontogeny matches the sequence of bar loss across evolution [46, 47]. In zebrafish, color pattern composed of periodic stripes forms through a reaction–diffusion system (Turing model), whereby stripe numbers depend on the size of the fish (review in [45]). In anemonefish, the developmental mechanism at the origin of the formation of the white bars differs, as the number of bars does not depend on the size of the fish [46, 47]. Moreover, in anemonefish, new bars do not form when the distance between two existing ones increases but are added following an ordered anterior-to-posterior pattern. Thus, a Turing-like model cannot solely explain the appearance and/or disappearance of bars during anemonefish ontogeny. This suggests that in anemonefish the temporal and spatial control of bars is influenced by specific patterning mechanisms that remain to be analyzed.

In zebrafish, it has been shown that cell–cell communication between the various types of pigment cells is instrumental in controlling pattern formation [45]. Whether similar mechanisms operate in the context of the antero-posterior color patterning in anemonefish is unclear. It has also been shown that the adult pigmentation pattern of zebrafish is controlled by thyroid hormones [48, 49]. As these hormones are known to control coral reef fish metamorphosis [50], it will be interesting to compare the gene regulatory programmes controlled by this hormone in the various pigment cells in zebrafish and anemonefish and to study how they may be implicated in color pattern diversification. Sex change does not lead to a profound modification of the pigmentation pattern in anemonefish, as described for example in Labrids [51]. However, in some species, minor changes in pigmentation have been observed in relation to the sexual status of individuals [12, 52].

### Phenotypic plasticity and genetic assimilation

One major question in Evo/Devo is whether phenotypic differences between related species may originate from genetic assimilation of preexisting phenotypic plasticity [53]. It has been observed that several anemonefish species (*A. clarkii*, *A. percula,* etc.) can adapt their body color to the species of sea anemone in which they live and that some isolated populations present naturally one of these alternative morphs (Fig. 3f, g) [54]. These cases provide very nice examples to study how environmental variations produce alternative phenotypes, and how these phenotypes may be fixed in some populations or species.

### Analysis of complex behavior

Sound production is widespread in vertebrate taxa and may be involved in evolutionary processes such as speciation, as demonstrated in insects, fish, anurans and birds [55]. In teleost fish, the ability to emit sound has been developed independently in more than 100 families [56]. Several lines of evidence suggest that sound communication in anemonefish is important for controlling social hierarchies [57, 58]. The genetic pathways controlling sound production and hearing, as well as the neural pathway controlling the processing of acoustic information and its effect on social relationships can therefore be analyzed in anemonefish.

Juvenile and adult anemonefish exhibit complex stereotyped behaviors associated with the social hierarchy as well as egg protection and sea anemone caring. Despite the importance of this behavioral complexity from an evolutionary ecological point of view, the development of neural structures and networks underlying such behaviors has been poorly studied at the cellular and molecular levels. Anemonefish would allow investigating the link between internal actors such as neurotransmitters, hormones, and behavioral responses. As their radiation is well understood, *Amphiprion* and *premnas* species also allow addressing the evolutionary origins of these complex behaviors.

## Experimental approaches

### Long term field experiments and monitoring

Anemonefish inhabit the same sea anemone during most of their lives: it is, therefore, possible to follow the same individual or the same anemonefish colony over time, at several month intervals, in the wild. It is also possible to make transplantation experiments, in which individuals are removed from one colony and introduced to a new one [59]. It is, therefore, possible to conduct long term studies monitoring the recruitment, changes in colony constitution and social organization, and sex transitions over several years [60, 61].

In Papua New Guinea, several teams from Australia, France, USA and Saudi-Arabia have teamed up to better understand the recruitment process in *A. percula*. Planes et al., [62] used microsatellites to fingerprint all breeding adults (125 fish) present on the isolated Kimbe island, in which 270 sea anemones have been precisely located [62]. Since the fish show strong fidelity to their sea anemone, they can be found at the same place after several months, except in the relatively rare case of mortality. Then, every two years, this team traveled to Kimbe to collect new recruits in the various sea anemone and determine who are their parents. Since all breeding pairs present in Kimbe were fingerprinted, they observed that 50% of new recruits are coming from outside the island, 8–10 km away. This demonstrated that *A. percula* larvae can effectively travel over long distances. Such a unique data set, accumulated over 10 years, also enabled to compare the survival of local recruits (e.g. whose parents come from Kimbe) vs. outsiders and to show the absence of trans-generational fidelity to sea anemone species [63, 64]. Because long term genealogical trees can now be constructed, this unique Kimbe dataset offers a great perspective to investigate the heritability of specific traits such as body size, pigmentation traits, etc.

### Behavioral experiments

Anemonefish offers a variety of behavior that can be studied both in the lab and in the field. First, it is easy in the lab to study the olfactory or visual perception of anemonefish larvae. The small size of larvae and the early development of their sensory abilities enable to follow the ontogeny of visual and olfactory perception and how it is used to locate sea anemone in the wild [65, 66]. Preliminary tests conducted by our group on swimming behavior also revealed that anemonefish larvae are suitable for digital time-lapse video recording. Such methods will facilitate detailed and well-replicated behavioral studies. Anemonefish may also allow conducting high-quality video recording for behavioral analysis in the wild as they remain in a small area.

### Pharmacological treatments of larvae

Functional studies are possible thanks to a method that allows to raise larvae and juveniles in a small volume (500 ml) (Roux et al., unpublished data). Pending the development of functional genetic methods, it is possible to use pharmacological approaches, using well-characterized drugs to alter specific biological pathways. One interesting asset here is the relative proximity of anemonefish with established fish models such as zebrafish and medaka, in which extensive chemical screens have been performed [67]. For example, our group has used TAE684, an inhibitor of tyrosine kinase receptors, *Ltk* and *Alk,* known to decrease iridophore number in zebrafish [68], to show that white bars in *A. ocellaris* are indeed formed by iridophores and allowed to obtain *A. ocellaris* juveniles devoid of white bars [47, 69].

### In situ* hybridization and multicolor fluorescence *in situ* hybridization (FISH)*

**Fig. 4:**
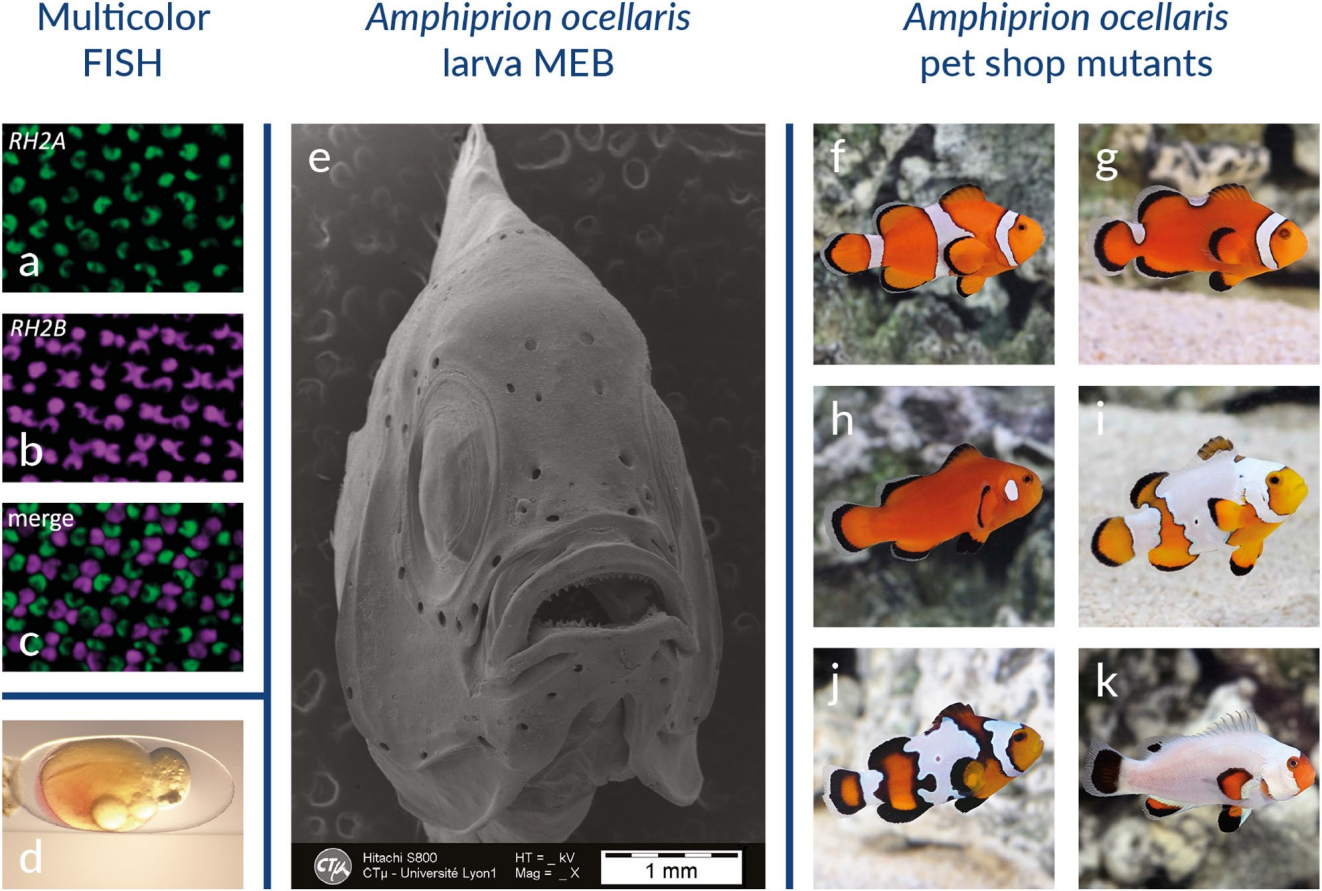
Experimental techniques commonly used in anemonefish. **a**–**c** Multicolor FISH in the retina, showing that *RH2A* (green) and *RH2B* (magenta) are expressed in opposite members of every double cone throughout the retina. Scale bar 10 µm.[72]. **d** Pictures of an embryo after successful injection of red dye. **e** Scanning electron microscopy of the larval head of *Amphiprion ocellaris*. **f**–**k** Pictures of wild-type (**f**) and color pattern mutants of *Amphiprion ocellaris* (**g**–**k**)

To follow the patterns of gene expression, in situ hybridization has been developed in anemonefish embryos [70] as well as other tissues such as gonads [71] and skin [69]. Those protocols are adapted from those established in zebrafish, taking into account the fact that anemonefish are marine species, therefore introducing additional washing steps to avoid salt precipitation. Multicolor fluorescence in situ hybridization (FISH) has enabled the simultaneous visualization and quantification of the expression of different opsin gene transcripts in the eyes of the Great Barrier Reef anemonefish *A. akindynos* (Fig. 4a–c) [72]. This has allowed the authors to reveal visual specializations and to understand the increase of chromatic contrast in this species, which may play an important role in detecting conspecifics.

### Scanning and transmission electron microscopy

New protocols for scanning and transmission electron microscopy have been specifically developed for marine fish to preserve tissue integrity and avoid osmotic issues due to salinity [73]. Such protocols have been successfully used to study the development of anemonefish organs such as teeth and sensory organs using scanning electron microscopy (Fig. 4e) [74, 75] and to determine the morphology of pigment cells in anemonefish skin using transmission electron microscopy [69].

### Pet shop mutants

Anemonefish are very popular in the aquarium trade and, not astonishingly, fancy mutants have been developed for trade purpose. Pet shops offer many color pattern mutants (Fig. 4f–k). As in other cases (e.g. zebrafish, zebrafinch) those mutants can be very useful scientifically as they allow addressing the developmental mechanisms at the origin of pigmentation pattern [45, 76]. Identifying the genes that underlie such deficient pigmentation pattern using Genome-Wide Association Studies (GWAS) will provide answers to fundamental questions such as how the border between the white, orange and black pigment cells is formed.

Wild anemonefish populations are also providing a large reservoir of pigmentation variants, from small variations in white bars shape to more extreme variants passing through the melanization plasticity described above. To our knowledge, there is up to now no general description of the extent of these variations.

### Natural hybrids

Hybridization is increasingly recognized as an important evolutionary process, especially in the emergence of evolutionary radiations, and this has also been shown to be important in anemonefish [77]. In that respect, it is important to mention that hybridization occurs frequently in the wild. Two species, *A. leucakranos* and *A. thiellei,* are in fact hybrids. The case of *A. leucokranos*, a hybrid between *A. chrysopterus* female and *A. sandaracinos* males, has been investigated to understand the evolutionary consequences of hybridization [78].

### Limitations

As with all experimental system, anemonefish have limitations. Perhaps the most obvious is the lack of established approaches for genetic manipulation. Several labs are now trying to develop gene editing using CRISPR/CAS9. No protocol has been published detailing how to inject anemonefish eggs, however, injection trials have been successful in our team with a survival rate of ca. 90% (illustrated on Fig. 4d). However, it must be made clear that the generation time of 9–18 months (depending on the species) impose some practical constraints on functional genetics approaches [79, 80]. The ability to generate F0 mosaic mutants should allow testing the functional importance of given genes in specific processes directly. Another limitation is that larvae are still very difficult to capture in the wild, making post-embryonic development in the wild inaccessible until the time of recruitment.

## Research community and resources

The anemonefish community, which has its origins in the ecology community, has been constantly growing and diversifying over the last 10 years as these fish have gained interest as experimental models. There are more than 10 research groups currently using anemonefish as a model for Evo/Devo or Eco/Evo/Devo, and most of these groups have established strong links with the ecology communities that study these fish in the field. This merging of communities, between ecology and evolutionary developmental biology, is extremely fruitful and will allow asking increasingly relevant questions and performing more integrated studies.

Every year, a “anemonefish day meeting” is organized by laboratories located in France, Japan, Switzerland, and Belgium that work on anemonefish.

The genomes of 11 *Amphiprion* and *Premnas* species have been sequenced (Table 1) and are available in the NCBI’s reference sequence database. Moreover, transcriptomes of 6 *Amphiprion* species have also been sequenced (Table 1). Most anemonefish species and their color pattern mutants are directly provided by pet shops. However, no standard strains are maintained and shared yet between the anemonefish research lab community. Moreover, a website providing information on the features, geographic distribution and images of each species is available (https://amphiprionology.wordpress.com). A community portal should be completed in the coming months, which will include protocols, pictures of adult and developing fish, organization of international meetings, and a community forum.

**Table 1:** Genomes and transcriptomes available for Amphiprion species research (*NA* Not available)

Species	Type of sequence	NCBI BioProject identifier	DOI-Reference
*Amphiprion ocellaris*	Genome	PRJNA407816	10.1093/gigascience/gix137 [81]
*Amphiprion ocellaris*	Genome	PRJNA515163	10.1093/gbe/evz042 [4]
*Amphiprion ocellaris*	Transcriptome male and female	PRJNA374650	NA
*Amphiprion ocellaris*	White and orange skins transcriptome	PRJNA482393	10.1111/pcmr.12766 [69]
*Amphiprion ocellaris*	Post Embryonic Developmental transcriptome	NA	10.1186/s12915-018–0559-7 [47]
*Amphiprion percula*	Genome	PRJNA436093	10.1111/1755–0998.12939 [82]
*Amphiprion percula*	Transcriptome of the brain	PRJEB27750	NA
*Amphiprion bicinctus*	Genome	PRJNA515163	10.1093/gbe/evz042 [4]
*Amphiprion bicinctus*	Transcriptome of brain and gonade of female, male and sex-changing individuals	PRJNA261388	10.1038/srep35461 [39]
*Amphiprion clarkii*	Transcriptome of the brain	PRJEB27750	NA
*Amphiprion frenatus*	Genome	PRJNA433458	10.1111/1755–0998.12772 [83]
*Amphiprion melanopus*	Genome	PRJNA515163	10.1093/gbe/evz042 [4]
*Amphiprion melanopus*	Transcriptome of gill	PRJNA398732	10.1186/s13742-016–0124-7 [84]
*Amphiprion polymnus*	Genome	PRJNA515163	10.1093/gbe/evz042 [4]
*Amphiprion sebae*	Genome	PRJNA515163	10.1093/gbe/evz042 [4]
*Amphiprion sebae*	Transcriptome of the brain of *Amphiprion Sebae* maturing stage	PRJNA285007	NA
*Amphiprion akallopisos*	Genome	PRJNA515163	10.1093/gbe/evz042 [4]
*Amphiprion nigripes*	Genome	PRJNA515163	10.1093/gbe/evz042 [4]
*Amphiprion perideraion*	Genome	PRJNA515163	10.1093/gbe/evz042 [4]
*Premnas biaculeatus*	Genome	PRJNA515163	10.1093/gbe/evz042 [4]

## Data Availability

Not applicable.
